# Issues and Applications in Label-Free Quantitative Mass Spectrometry

**DOI:** 10.1155/2013/756039

**Published:** 2013-01-16

**Authors:** Xianyin Lai, Lianshui Wang, Frank A. Witzmann

**Affiliations:** ^1^Department of Cellular & Integrative Physiology, Biotechnology Research & Training Center, Indiana University School of Medicine, Indianapolis, IN 46202, USA; ^2^School of Informatics and Computing, Indiana University, Bloomington, IN 47408, USA

## Abstract

To address the challenges associated with differential expression proteomics, label-free mass spectrometric protein quantification methods have been developed as alternatives to array-based, gel-based, and stable isotope tag or label-based approaches. In this paper, we focus on the issues associated with label-free methods that rely on quantitation based on peptide ion peak area measurement. These issues include chromatographic alignment, peptide qualification for quantitation, and normalization. In addressing these issues, we present various approaches, assembled in a recently developed label-free quantitative mass spectrometry platform, that overcome these difficulties and enable comprehensive, accurate, and reproducible protein quantitation in highly complex protein mixtures from experiments with many sample groups. As examples of the utility of this approach, we present a variety of cases where the platform was applied successfully to assess differential protein expression or abundance in body fluids, *in vitro* nanotoxicology models, tissue proteomics in genetic knock-in mice, and cell membrane proteomics.

## 1. Introduction

Protein quantification for differential expression analysis or expression profiling represents the most challenging aspect in proteomics technology. This task is typically carried out through array-based [1], two-dimensional-electrophoretic (2-DE-) based [2] or mass-spectrometry- (MS-) based approaches [3, 4]. MS-based approaches are normally referred to as “bottom-up” rather than “top-down,” because the top-down approach has not yet reached its full potential. In bottom-up quantitative approaches, complex protein mixtures are digested enzymatically, peptides from each protein are separated by liquid chromatography (LC) and detected by MS, and protein quantification is completed at the peptide level and then combined to calculate a summarized value for the protein from which they come. Early in the evolution of quantitative MS-based proteomic technology, stable isotope labeling methods were developed [5–8]. Following that premise, many new label-based methods have arisen. However, all of these suffer from several limitations: (i) additional sample processing steps in the experimental workflow, (ii) high cost of the labeling reagents, (iii) variable labeling efficiency, and (iv) difficulty in analyzing low-abundance peptides in multiple samples, especially when numerous experimental groups are studied [9].

**Figure 1:**
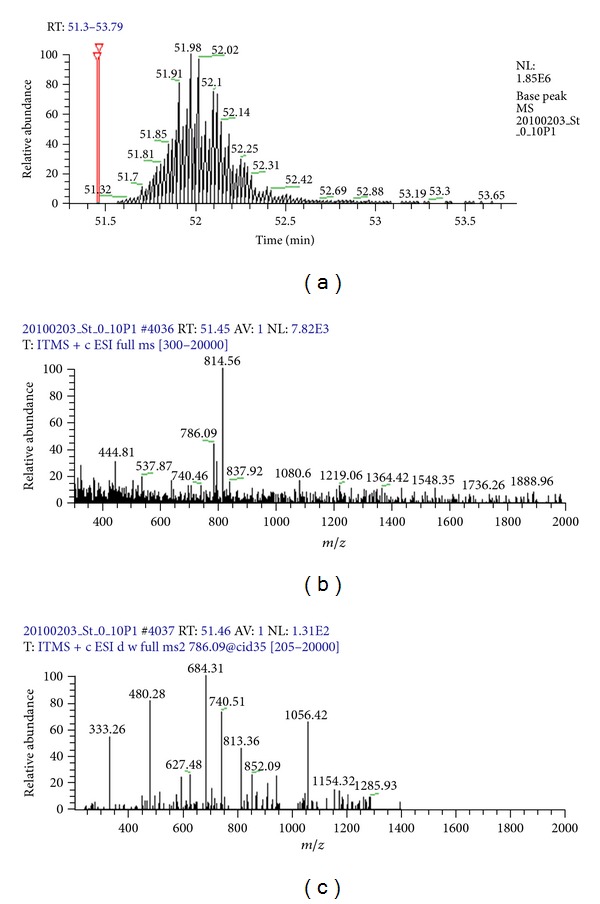
A typical LC-MS/MS analysis of a peptide. (a) The peptide is eluted from an LC column and its ion intensity is recorded at different time points, forming a peptide peak. The scan time points for (b) and (c) are labeled in red. (b) At scan #4036, a full MS scan is performed, and all the peptide ions including ion *m/z* 786.09 are recorded. (c) At scan #4037, an MS/MS scan is performed, and the ion *m/z* 786.09 is chosen as a precursor ion to generate product ions, providing peptide fragmentation information for peptide identification.

**Figure 2:**
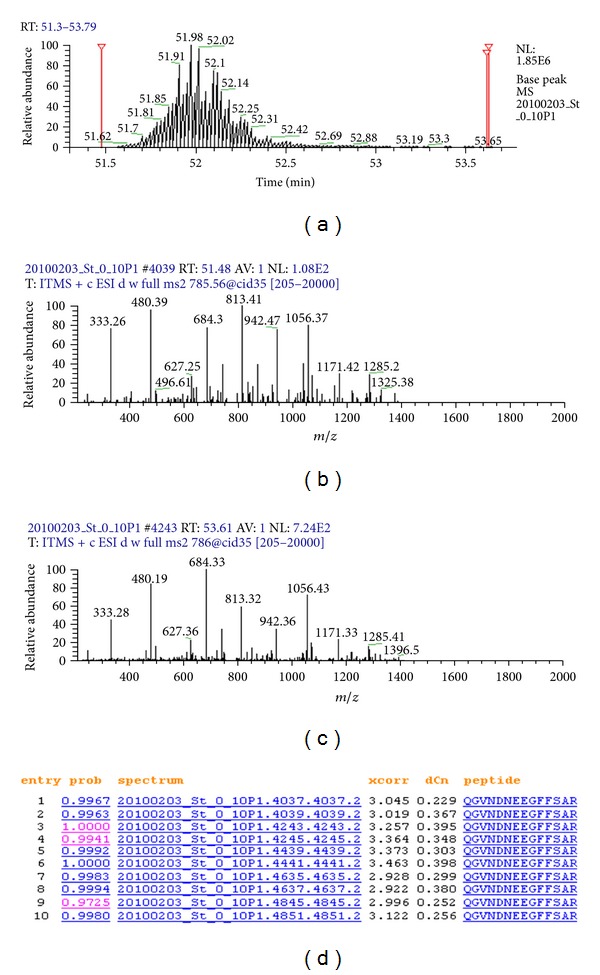
Multiple MS/MS scans of a single peptide. (a) The peptide is eluted from an LC column. The scan time points for the last MS/MS scan before the peak and the first two MS/MS scans after the peak are label in red. (b) At scan #4039, an MS/MS scan of the precursor ion *m/z* 786.09 is performed almost one-half minute before the apex of the peptide peak. (c) At scan #4243, the first repeat MS/MS scan of the precursor ion *m/z* 786.09 appears two minutes later and is performed almost one-and-a-half minutes after the apex of the peptide peak. (d) All the MS/MS scans of the peptide are listed. Most of them appear far behind the peptide peak.

Following the development of label-based approaches, label-free approaches emerged to overcome the drawbacks associated with label-based approaches mentioned above. To illustrate the principles of label-free quantitative mass spectrometric (LFQMS) approaches, we present the following example. Along with the elution (retention time) of a peptide from an LC column (Figure 1(a)), the peptide peak height (intensity) and mass-to-charge (*m/z*) are recorded in a mass spectrum (Figure 1(b)), the peptide ion may be chosen as a precursor ion at some specific time point according to specific parameter settings to generate an MS/MS spectrum where intensity and *m/z* of the product ions in the MS/MS spectrum are recorded (Figure 1(c)). The peptide ion may be chosen as a precursor ion multiple times (Figures 2(a)–2(d)). From the LC chromatogram, MS spectrum, and the MS/MS spectrum, all information associated with peptide abundance, such as the peptide peak intensity (height or area of a peak), the peptide precursor ion peak height, and the peak height of product ions, can be extracted. Using such information individually or combinatorially, numerous label-free methods have been developed, including two extensively applied but fundamentally different strategies: quantitation based on spectral counting [10] and peptide ion peak area [11].

Spectral counting estimates protein abundance by counting the number of spectra matched to peptides from a specific protein. In the example shown in Figures 1 and 2, ten MS/MS spectra were acquired for a peptide precursor ion, but they were acquired either before or after the actual peptide elution peak. Data acquisition in this manner is most typical when the dynamic exclusion mode is applied to identify substantially more peptides. In this case, it is erroneous to hypothesize that peptide abundance is correlated with the number of spectra. Although spectral counting has been applied to study differential protein composition in complex biological samples, when low-end MS and limited LC separation (such as one-dimensional LC) are applied, protein quantification using spectral counting is challenging because (i) dynamic exclusion of ions during data acquisition to obtain more MS/MS fragments of low-abundance peptides dramatically and adversely affects spectral acquisition, and (ii) coeluting peptides compete for MS/MS analysis and influence spectral acquisition [9]. In contrast, these issues are less consequential when peptide peak area is used to measure peptide abundance.

Peak area has been applied extensively in the quantification of small molecule compounds [12–14] and is the most reliable measurement for quantification. Because peptides perform similarly to small molecule compounds in liquid chromatography-tandem mass spectrometry (LC-MS/MS) analysis, direct measurement of peak area is considered a reliable method for peptide and protein quantification. Numerous software packages for label-free quantification of LC-MS/MS-derived data based on peptide peak area have been developed and applied in proteomics research. However, peptide and protein quantification is not as straightforward as quantification of small molecule compounds, because thousands of peptides are analyzed, identified, and quantified in a single run as compared to small molecule analysis, where the focus is only on one or a few small molecule compounds. To accurately quantify peptides and proteins, several issues must be addressed. This paper will present these issues, describe ways in which to address them, and provide examples where comprehensive quantitation has been carried out successfully.

## 2. Issues

### 2.1. LC-MS Alignment

**Figure 3:**
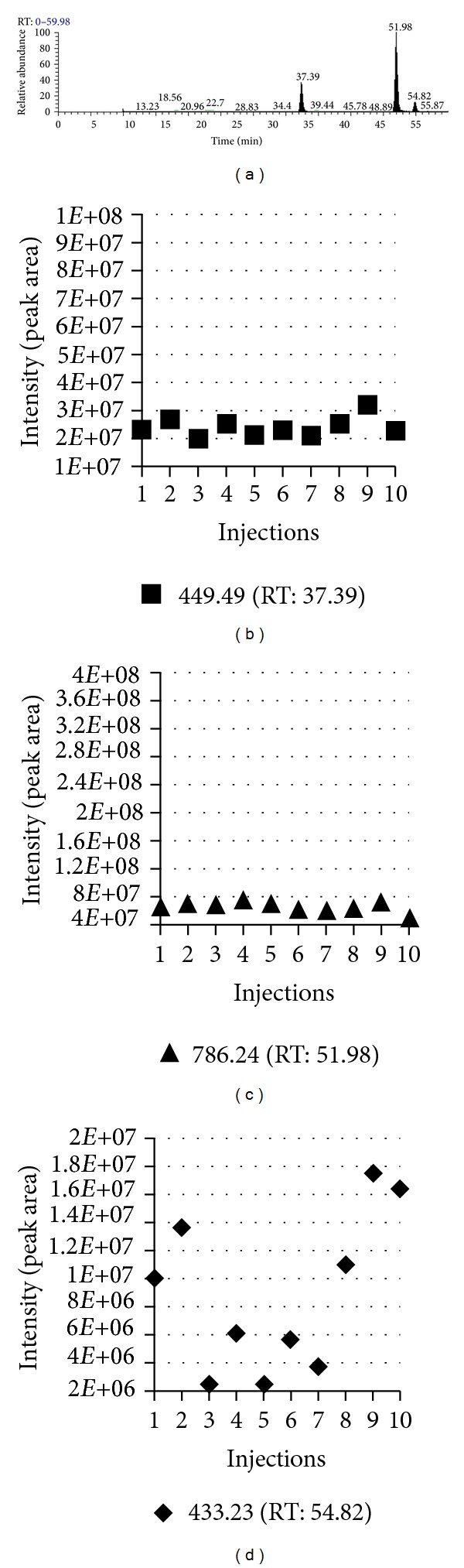
A typical LC-MS chromatogram of three standard peptides. (a) 4 pmol of Angiotensin III, Fibrinopeptide B, and Angiotensin I were mixed and analyzed by LC-MS/MS 10 times. (b–d) Intensity of Angiotensin I, Fibrinopeptide B, and Angiotensin I at mean *m/z* 449.49, 786.24, and 433.23 Da from 10 injections was calculated, respectively. Their CVs are 15.0%, 11.4%, and 63.4%, respectively. Also, Figures (b–d) show that identical amounts of peptides generate totally different peak area, and the fold difference is as large as 7.45 (6.63*E* + 07/8.90*E* + 06).

To extract the peptide peak area, two basic parameters, *m/z* and retention time, must be determined. Typically, the *m/z* value is measured reproducibly in low resolution mass spectrometers such as the LTQ and extremely reproducibly in high resolution mass spectrometers such as LTQ-Orbitrap. In general, we have consistently observed that the retention time of each peptide in LC normally varies by roughly 3 min [9]. When dynamic exclusion is enabled, the MS/MS scan of a peptide does not normally occur at the peptide elution peak. For example, a typical LC-MS chromatogram of three standard peptides is shown in Figure 3(a). The *m/z* value variations of three standard peptides from 10 injections are 0.36, 0.89, and 0.26 Da for Angiotensin I, Fibrinopeptide B, and Angiotensin I at mean *m/z* 449.49, 786.24, and 433.23 Da, respectively. Their retention time variations are 2.06, 1.81, and 1.82 min at mean retention time 38.76, 53.26, and 56.08 min, respectively. As shown in Figures 1 and 2, the MS/MS spectra appear at least 0.5 min before the apex of the elution peak and 1.6 min after the apex.

As the results above indicate, chromatographic alignment of peptide elution peaks can be a significant issue in certain cases. Thus, it is important to determine if alignment is necessary. For instance, when high resolution mass spectrometry is applied, peptides are completely separated in the *m/z* dimension, forming individual peaks. If the *m/z*, retention time, and ID of each peptide peak are extracted from an individual sample, the comparison of multiple samples does not necessarily require alignment. If comparison of multiple samples is carried out using a workflow similar to that used in 2-DE image analysis [15, 16], alignment is required. However, when low resolution mass spectrometry is applied, peptides cannot be separated completely in the *m/z* dimension, and they rarely form individual peaks. The apex of the peptide elution peak is thus difficult to determine. Using MS/MS spectral time instead of apex time to extract peptide peak area from individual samples is doable, but alignment of multiple samples enables more accurate estimation of peptide elution retention time, leading to more accurate quantification.

A second issue relates to when it is best to perform the alignment. Should it be performed before or after peptide identification? In the development of label-free quantification techniques, researchers initially followed a workflow similar to that used in 2-DE image analysis, for example, aligning each peptide from different samples by plotting them as two-dimensional images [17]. In 2-DE gel image analysis, information regarding protein identity is unavailable. Consequently, using resolved proteins as landmarks to align images is the best and only option, although it is likely that protein “spots” arising from dissimilar proteins may be aligned as the same protein. However, in an LC-MS/MS analysis, information regarding peptide identity can be obtained easily so that every identified peptide can serve as a landmark, making thousands of landmarks available. Therefore, to improve quantitative accuracy, alignment after peptide identification should be carried out.

The third issue regarding alignment relates to how the alignment should be carried out. Currently, five approaches typically are used to align the retention time of peptides across multiple injections. The most popular alignment is similar to that used in 2-DE image analysis, such as OpenMS/TOPP [18], MapQuant [19], Msight [15], msInspect [20], and MZmine [21]. Enhancement of the 2-DE mode alignment is accomplished by adding peptide ID information to improve alignment accuracy, in programs such as SuperHirn [22] and PEPPeR [23]. A third approach is to align the “Base Peak” chromatograms, as performed by SIEVE from Thermo Scientific, and a fourth approach is an alignment using MS/MS scan times (identified peptides), such as that found in IDEAL-Q [24].

We have developed an alternative approach to those above, IdentiQuantXL, which uses individual three-dimensional alignment to determine peptide retention time using a clustering method [9]. Approaches 1 and 2 perform well only for high resolution data. However, for low resolution data, these approaches are incapable of generating analyzable patterns. SIEVE was designed for both high and low resolution data, aligning the Base Peak chromatogram via Recursive Base-Peak Framing to generate a unique “frame” for each group of peaks within a specified *m/z* and retention time range. However, when low-resolution data are analyzed, SIEVE performs poorly for the following reasons. First, this approach ignores the fact that the retention time variation of each peptide is not consistent with that of the Base Peak. Second, it is difficult for SIEVE to align two dissimilar distributions and align to a void. Finally, the “frame” (a rectangular region containing *x* and *y* coordinates in the *m/z* versus retention time plane) defined by SIEVE is too subjective. Because SIEVE generates “frames” with only a one-size time-width, its application in complex samples where peak-widths are variable is not practical. Even when the same sample is injected twice, specific peptide peaks will have variable widths. IDEAL-Q uses a linear regression model to determine the retention time of each peptide. However, it ignores the fact that most peptides have multiple MS/MS spectra and appear at different time points, generating one of the six elution patterns observed by Lai et al. [9]. Only IdentiQuantXL considers all these issues and performs an individual, three-dimensional (*m/z*, RT, and MS/MS ID) alignment to determine peptide retention time using a clustering method, providing the most accurate retention time determination and widest application. 

In IdentiQuantXL, all MS/MS scan times of a peptide are collected, a scan time distribution pattern is generated, a cluster is applied when the scan time range is over 3 min, and the weighted mean scan time is determined as the peptide retention time. In this method, the alignment is processed using the MS/MS scan times to ensure that only the same peptides are aligned across injections. Because most peptides have multiple MS/MS spectra and appear at different time points, the clustering approach is better able to determine peptide peak retention time accurately.

### 2.2. Qualified Peptides for Protein Quantification

When peptides in a complex sample are analyzed by LC-MS/MS, their behavior in each component of the analytical platform is diverse, a phenomenon that largely has been ignored. First, peptides are unlikely to be identified in every injection. Because of proteomic sample complexity, tens of thousands of peptides may exist in a sample. Only the most highly abundant peptides are likely to be selected for MS/MS analysis in each injection. Frequently, an individual peptide's intensity may be higher or lower than other coeluted peptides in various injections, leading to selection for fragmentation and subsequent identification of different peptides in different injections. For example, when 35 injections of a tryptic digest of whole rat kidney lysates were analyzed, generating 7,361 peptides [9], 25.6% of the peptides (1,883) were identified in 1 injection and only 0.8% of the peptides (61) were identified in all 35 injections. If a peptide is not analyzed by MS/MS in an injection, that does not mean it is absent from this injection. A peptide identified only in one injection may exist in all other injections in that experiment. However, if the once-identified peptide is a misidentified peptide, extraction of this peptide from all other injections leads to erroneous quantification. The use of peptides with low identification frequency for quantitation constitutes a risk of error, but no clear cutoff exists to exclude such peptides. The higher a peptide's identification frequency, the greater confidence one can have that the peptide actually exists in all the injections.

Second, peptides have multiple elution patterns across various runs in a typical experiment. For a complex sample, a single chromatographic condition, for example, one specific column with specific mobile phases and gradient, cannot be optimal for each of the thousands of peptides in a single sample injection. Six elution patterns have been summarized [9]: (i) the peptide is completely eluted at one time point and very consistent in different sample injections analyzed; (ii) it is completely eluted at one time point but not consistently in all injections; (iii) its abundance is very high in some injections and cannot be bound completely to the column matrix, so some amount of the peptide is eluted before the main peak; (iv) it is bound to the column matrix too tightly, eluting mainly at first, with a remainder eluting at higher organic concentration, after the main peak; (v) it has an overall elution pattern combining patterns (iii) and (iv); and (vi) the peptide has poor chromatographic performance and is eluted at indistinct time points across all injections. Consequently, some peptides having an elution pattern like (vi) do not form a peak, precluding quantitation.

Third, the peak area coefficients of variation (CVs) of peptides from two injections of one sample are highly variable. Due to the fact that chromatographic conditions cannot be optimal for all of the thousands of peptides detected in a single injection, some peptides have a very small CV, while other peptides have a very large CV. For instance, a comparison of the intensities of 4 pmol of the mass standards Angiotensin III, Fibrinopeptide B, and Angiotensin I revealed that their CVs were 15.0%, 11.4%, and 63.4%, respectively [9] (Figures 3(b)–3(d)).

Finally, when multiple peptides are used to calculate a protein's abundance in a comparative study, individual peptides often exhibit fold changes that are different from other peptides from the same protein. Several explanations for this phenomenon are possible: (i) some peptides have greater variation than others under the same chromatographic conditions; (ii) posttranslational modification (PTM) variably affects the relative abundance of unmodified peptides; (iii) peptide sharing among diverse proteins causes inconsistent effects on some peptides; (iv) carry-over of some peptides causes random abundance changes, and (v) differential regulation of isoforms, misidentification, and misquantification also may occur [9, 25].

From the discussion above, a fundamental concept emerged that not every peptide can be used to quantify a corresponding protein. Unquantifiable peptides are caused by limitations in mass spectrometry, uniform (not individually optimized) chromatographic conditions, PTMs, peptide sharing, and so forth. These issues normally cannot be addressed prior to data generation, and, currently, there are no proper means to correct them. One practical way is to eliminate the unquantifiable peptides before they are used for protein quantification. Lai et al. have developed multiple filters (peptide frequency, retention time, intensity CV, and correlation) to specifically address this issue and enhance the accuracy of protein quantification [9].

### 2.3. Normalization

The aim of normalization is to remove systematic bias. It was first used in the analysis of gene expression data obtained from laser or optical microarrays and then applied to label-free quantification in high-throughput proteomics [26]. Numerous normalization algorithms have been developed and applied. Among them, global normalization (central tendency), linear regression, local regression, and quantile techniques are the commonly used methods for normalization of peptide abundance measurements obtained from LC-MS.

When global normalization is applied, it is assumed that every peptide produces the same peak area at the same abundance. However, this is not the case in proteomic analysis. For example, when three standard peptides were injected in identical amounts (Figures 3(b)–3(d)), LC-MS generated totally different peak areas, and the fold difference was as large as 7.45 (6.63*E* + 07/8.90*E* + 06).

Linear regression normalization is applied to remove systematic bias caused by sample carry-over on an LC column [26]. However, in a typical experiment where multiple samples are compared, a blank is normally introduced between samples to wash the column and eliminate carry-over. Furthermore, even if minor carry-over occurs, it typically involves just a few peptides, not all of them [27].

Local regression normalization is used to eliminate systematic bias generated from the effects of ion suppression on measured peptide abundances, or on measured peptide abundances approaching detector saturation or background [26]. Again, these situations only happen to a few peptides, not every peptide. It is difficult to determine which peptides are suitable for this normalization. 

Quantile regression normalization is employed under the assumption that the distribution of peptide abundances in different samples is expected to be similar and can be accounted for by adjusting these distributions [26]. However, in an actual experiment, peptide abundances in samples from different groups or even the same group differ because of biological variability or experimental manipulation or treatment.

Normalization using singly and multiply spiked internal standards has been reported [24, 28]. This strategy is not new and has been applied in pharmaceutical analyses for many years. Isotopic labeled standards, the multiple reaction monitoring (MRM) technique, and highly optimized LC conditions are applied in this strategy to obtain specific internal standard peaks. However, tens of thousands of peptides may exist in a single sample, and their peak area response with their abundance may be significantly different. Several internal standards are incapable of representing all the peptides and are thus of marginal value.

The systematic biases considered by some researchers include protein degradation, sample preparation, variation in sample loaded, and measurement errors [26, 29]. However, these biases are not compelling enough to warrant normalization. In proteomics experiments, proteins are denatured, reduced, and alkylated. Protein degradation rarely occurs during careful sample preparation when sample preparation is conducted by skilled technicians. Each sample is injected by autosampler with a high degree of accuracy and precision. When an appropriate normalization method is not available, the process of normalization is unnecessary and should be avoided, as it may introduce new errors in quantification.

Alternatively, use of sample quality control (QC) is a better way to obtain accurate results. QC samples and experimental samples should be analyzed in the same batch. If the QC samples indicate the instrument and running conditions are optimal, the data obtained from experimental samples can be considered valid and the systematic biases considered minor.

As an example of this concept, in an LFQMS analysis where none of the normalization techniques was applied [9], suitable precision was achieved, based on the acceptance criteria of assay performance by which biomarker assays are evaluated, CV ± 25% acting as the default value (±30% at the lower limit of quantitation, LLOQ) [30]. In a repeatability test of a simple 3 peptide sample, two quantifiable peptides (Angiotensin III and Fibrinopeptide B) had an intensity CV ≤ 15%. In the repeatability test of a more complex sample (kidney tissue), 772 (76.4%), 99 (9.8%), 102 (10.1%), and 38 (3.8%) out of 1,011 proteins had a CV ≤ 15%, ≤20% and >15%, ≤30% and >20%, and ≤50% and >30%, respectively, indicating that 96.2% of proteins had CVs ≤ 30%. These data indicate that elimination of unquantifiable peptides is more vital to accurate quantitation than normalization. In current LC-MS technology, no ideal normalization techniques exist. Using inappropriate or even flawed normalization will not improve the analysis and may introduce additional errors, thus it is better that no normalization is applied. However, filtration of unquantifiable peptides is absolutely necessary for an accurate analysis.

## 3. Applications

The true test of the utility of an LFQMS platform lies in the comparative analysis of differential expression in complex protein mixtures, that is, cell and tissue lysates or body fluids, across several experimental groups. In the following examples, we demonstrate the applicability of the IdentiQuantXL platform in overcoming many of the limitations of bottom-up quantitative approaches mentioned above to provide comprehensive and accurate analysis of protein abundance and differential expression in a range of experimental situations.

### 3.1. Aqueous Humor Proteomics

In the initial test of a beta version of IdentiQuantXL, we analyzed the protein composition of aqueous humor (AH) to investigate the role it might play in Fuchs endothelial corneal dystrophy (FECD) [31]. AH is the biologic fluid in the anterior chamber of the eye that protects and supplies nutrients to the cornea, lens, and trabecular meshwork. A balance between production and drainage of AH is critical to maintaining normal intraocular pressure, and its protein composition has been shown to change dramatically in various ocular conditions. Albumin and immunoglobulin G (IgG) depleted samples were obtained from male and female patients with and without FECD, and both depleted AH and albumin-bound proteins were analyzed. We identified 64 nonredundant AH proteins, most of which were identified in previous AH proteomic studies of patients with cataracts, in the albumin-depleted fraction. The levels of five of these were significantly lower (afamin, complement C3, histidine-rich glycoprotein, immunoglobulin heavy [IgH], and protein family with sequence similarity 3, member C [FAM3C]), while the levels of one, suprabasin, was significantly higher in patients with FECD compared to controls (*P* ≤ 0.01). We also identified 34 proteins in the albumin-bound fraction, four of which were significantly elevated in patients with FECD including a hemoglobin fragment, immunoglobulin kappa (IgK) and immunoglobulin lambda (IgL) (*P* ≤ 0.01). Additionally, we were unable to detect any significant differences in protein levels based on gender. Because FECD is a progressive disorder, regression analyses were performed to determine any significant correlations with age, and of interest retinol-binding protein 3 was significantly correlated with age in patients with FECD (*P* ≤ 0.01), whereas no proteins in the control group correlated with age. This was the first report indicating alterations in the AH proteome with FECD, demonstrating the utility of the LFQMS approach.

In a related study [32], we investigated the effect of implantation of a glaucoma shunt device on inappropriate accumulation of plasma-derived proteins in the AH. We identified 135 nonredundant proteins in the albumin-depleted fraction, 13 of which differed significantly between shunted and control groups. Those proteins play a role in oxidative stress, apoptosis, inflammation, and/or immunity. Many of the identified proteins were novel proteins not previously associated with glaucoma. All but complement C4 were known plasma proteins and the elevated levels of these proteins in the aqueous humor suggested that the glaucoma shunt device caused either a breach in blood-aqueous barrier or chronic trauma, increasing influx of oxidative, apoptotic, and inflammatory proteins that could potentially cause corneal endothelial damage.

### 3.2. Nanoparticle Exposure in Biological Systems

To assess the effect of functionalized carbon nanotube (f-CNT) and silver nanoparticle (AgNP) exposures, LFQMS was used to generate differential protein expression profiles in a novel *in vitro* intestinal cell model that features Caco-2 and HT29-MTX cells in coculture. These adenocarcinoma cell lines are derived from intestinal absorptive and mucus-secreting goblet cell types, respectively. Their combination and unique utilization represent a physiologically relevant GI model system characterized by tight junctions, polarity, and mucus secretion covering the entire monolayer. In a related study, we used LFQMS to compare the composition of proteins in coronas that form around f-CNT and AgNP in cell culture media supplemented with fetal bovine serum. As described below, the results of both of these various studies, in which the IdentiQuantXL platform was the key analytical tool, provide excellent examples of the utility of this platform in nanotoxicology. 

#### 3.2.1. Nanotoxicoproteomics of Functionalized Carbon Nanotube Exposure

To assess the biological effects of low level, water dispersible, functionalized carbon nanotube (f-CNT) exposure in the Caco-2/HT29-MTX coculture model (75% Caco-2, 25% HT29-MTX), cell protein expression was quantified and compared [33] using the IdentiQuantXL platform. Cocultured cells were exposed to well-characterized carboxylated single-walled carbon nanotubes (SWCNT-COOH), carboxylated multiwalled carbon nanotubes (MWCNT-COOH), and poly(vinylpyrrolidone) (PVP) polymer functionalized multiwalled carbon nanotubes (MWCNT-PVP) for 48 h at 500 pg/mL and 10 *μ*g/mL. The identification of 8,081 peptides (from technical replicates of *n* = 5 samples; 70 injections total) led to 4,743 protein database hits that were globally identified with >90% confidence, corresponding to 2,282 unique, nonredundant proteins that were compared across the dose groups. Among these, 428 proteins were differentially expressed (*P* < 0.01). As listed in Table 1, mean CV across the 2,282 proteins was less than 17% in all samples except SWCNT-COOH 500 pg/mL and MWCNT-PVP 10 *μ*g/mL where considerable differential expression was observed. Control group CV averaged only 12%. At the high dose, the extent of differential protein expression was CNT-specific and directly related to CNT colloidal stability. Surprisingly, cells responded to low level MWCNT-PVP exposure with 3-fold greater differential expression than at the high level. Bioinformatic analysis indicated significant and CNT-specific effects on relevant molecular and cellular functions and canonical pathways, with little overlap across CNT type and in the absence of overt toxicity.

#### 3.2.2. Nanotoxicoproteomics of Silver Nanoparticle Exposure

Analysis of 20 nm AgNP-citrate exposed to Caco-2/HT29-MTX cells for 3 and 24 h at 50 *μ*g/mL revealed significant alterations in cellular protein expression. A total of 4,112 unique proteins, homologs, or splice variants were identified, quantified and their abundance statistically compared. The identified proteins (from technical replicates of *n* = 3 samples; 24 injections total) corresponded to 13,445 identified peptides and 8,343 protein database hits, many of which were redundant. As listed in Table 2, mean CV across the 4,112 proteins was less than 20% in AgNP-treated samples and less than 17.3% in the controls. In fact, the number of proteins with CV ≤ 15% was 55% and 46% in 3 h and 24 h controls, respectively, and 38% and 45% in 3 h and 24 h AgNP-exposed groups, respectively.

**Figure 4:**
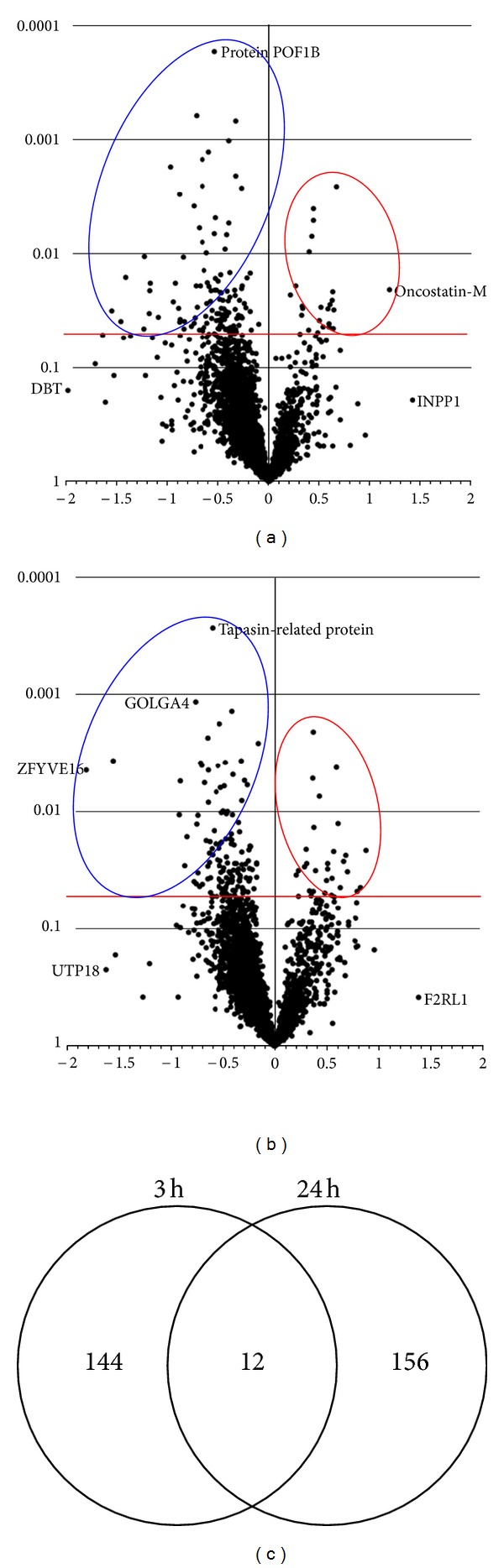
Volcano plots of all 4,112 Caco-2/HT29-MTX cell proteins with Log2 fold-change in expression on the *x* axis and *P* value on the y axis. (a) 20 nm AgNP-citrate 12.5 *μ*g/mL, 3 h effect. (b) 20 nm AgNP-citrate 12.5 *μ*g/mL, 24 h effect. Blue ellipses surround significantly decreased proteins; red ellipses are increased proteins. (c) Venn diagram illustrates that AgNP-mediated effects are unique at the two time points, and little overlap in biological effect occurs.

The 3-hour exposure significantly altered the expression of 156 proteins (130 decreased, 26 increased) while 168 (137 decreased, 31 increased) were differentially expressed after 24 h. Volcano plots in Figures 4(a) and 4(b) broadly illustrate the proteome response. Consistent with our previous NP studies, protein expression altered at early time points is different from that occurring in longer exposures. As the Venn diagram indicates (Figure 4(c)), clear differences in cellular response at these time points emerged. At 3 h, proteins associated with protein synthetic processes, ubiquitination pathways, and EIF2 signaling pathways were downregulated, as were those in the glycolytic/gluconeogenic pathway. These changes indicate events that result in a decline in global cellular protein synthesis. At 24 h, proteins of the glycolytic/gluconeogenic pathway are no longer affected, while proteins associated with cellular infection are upregulated.

When one compares the number of proteins identified and quantified in this study to the f-CNT study described in Section 3\tmspace +\thinmuskip {.1667em}3.2\tmspace +\thinmuskip {.1667em}3.2.1, it is apparent that significantly more proteins were evaluated in the AgNP experiments. Indeed, two-thirds of peptides were detected, leading to an 80% increase in the number of nonredundant proteins that were identified and quantified (4,112 versus 2,282). The difference lies in the ratio of Caco-2 to HT29-MTX cells in coculture and the duration of cell culture. In the f-CNT study, the ratio was 75 : 25 whereas in the AgNP study we found a ratio of 90 : 10 to improve the cell monolayer stability and integrity. Decreasing the percentage of mucus-secreting HT29-MTX cells significantly reduced the amount of mucus present when cells were recovered for sample preparation, and this enabled a proportionally higher yield of cellular proteins in the sample. A more minor consideration relates to the fact that f-CNT exposures were carried out for 48 h, compared to 3 and 24 h in the AgNP study, a point at which protein synthesis, growth, and proliferation are comparatively lower. Thus, the difference may be a function of longevity.

Similar successful applications of this platform to nanoparticle exposure in other cell culture systems have been conducted and can be found in the literature [34–36]. 

#### 3.2.3. Nanoparticle Protein Corona Composition

The protein corona (PC) that forms on nanoparticles when they are exposed to protein-containing biological fluids changes its characteristics and may play a significant role in nanoparticle bioactivity in cells [37]. Based on our own previous observations [33, 38] that structurally similar nanoparticles can have divergent biological effects in cell culture systems, we used the IdentiQuantXL platform to investigate the composition of the PCs formed on different high aspect ratio nanoparticles (nanoclay tubes, SWCNT-Raw, SWCNT-COOH, MWCNT-Raw, MWCNT-Pure, MWCNT-COOH, and MWCNT-PVP) mentioned earlier in this paper [39].

Proteomic analysis identified and quantified 366 different protein components of the various NT coronas. The PC which formed on the nanoclay tubes consisted of the fewest number, 82 different proteins, whereas the SWCNT-COOH corona contained the most, at 181. For reference purposes, analysis of the 10% FBS-DMEM media alone revealed 2,507 nonredundant proteins, polypeptides, or protein fragments/isoforms, and a list of these along with their peptide sequence and abundance data is provided along with the published manuscript [39].

All NT coronas were found to consist of 14 common proteins, including alpha-1-antiproteinase, alpha-2-HS-glycoprotein, alpha-S1-casein, apolipoprotein A-I, apolipoprotein A-II, keratin, type I cytoskeletal 10, keratin, type I cytoskeletal 15, keratin, type II cytoskeletal 1, keratin, type II cytoskeletal 5, keratin, type II cytoskeletal 6A, keratin, type II cytoskeletal 6C, keratin, type II cytoskeletal 75, serum albumin, and titin. The five most abundant coronal proteins (titin, serum albumin, apolipoprotein A-I, apolipoprotein A-II, and alpha-S1-casein) exhibited significant differences across the various NTs, while the relative contributions of alpha-1-antiproteinase (aka alpha-1-antitrypsin in humans), alpha-2-HS-glycoprotein, and the 7 keratins to the NT coronas were not significantly different. With the exception of titin, alpha-S1-casein and the keratins, the highly abundant serum proteins are commonly found in NP coronas formed in human plasma/serum. Titin is the 14th most abundant protein in the FBS-DMEM media whereas albumin is 1st, alpha-2-HS-glycoprotein 2nd, and alpha-1-antiproteinase 3rd, and Apo-AI is 17th, while alpha-S1-casein and the keratins (other than keratin 1) are far less abundant in the culture medium. Importantly, the presence of the latter proteins in the PC of all NPs suggests a selective enrichment that is not related to their concentrations in the media. It should also be mentioned that all of the above proteins are highly abundant in human plasma according to the most recent version of the Human Peptide Atlas database (http://www.peptideatlas.org), with the exception of alpha-S1-casein, which is not a component of human plasma [40].

The most abundant PC protein was Xin actin-binding repeat-containing protein 2 (XIRP2) and it was found only in MWCNT-Pure, MWCNT-PVP, and SWCNT-COOH coronas. XIRP2, aka mXin*β* and myomaxin, is a 382,300 Da protein expressed in cardiac and skeletal muscle where it interacts with filamentous actin and *α*-actinin through the novel actin-binding motif, the Xin repeat [41, 42]. It is also the 40th most abundant protein in the FBS-supplemented culture medium. Like titin, this largely abundant coronal protein is associated with intracellular filamentous proteins. The ample presence of XIRP2 in the media and in NT coronas may be in the form of protein fragments that are more common to fetal serum and less so in adult human or bovine sera where they are known to interact with albumin [43]. Other proteins may also be present in the PC via their association with bovine serum albumin, as part of the albuminome [43–45]. For instance, the keratins identified in the PCs may be there through their interaction with albumin directly, or indirectly via their known interaction with apolipoproteins, which also interact with albumin [46]. The results above represent one of the most comprehensive analyses of both protein corona constituents and the protein composition of fetal bovine supplemented culture medium, underscoring the utility of the LFQMS platform.

### 3.3. Pituitary Proteomics

As part of an investigation of the molecular differences between wild-type (WT) and *Lhx*3^*W*227*ter*/*W*227*ter*^ knock-in mice (a model of pediatric combined pituitary hormone deficiency (CPHD) disease), we conducted a quantitative proteomic analysis of the proteins expressed by adult pituitary glands [47]. In 100–200 *μ*g tissue samples (from which 20 *μ*g of peptides were injected), a total of 2,416 nonredundant proteins were identified and quantified from 7,680 unique peptides representing 4,352 protein database hits. Coefficients of variation across all 2,416 proteins in WT and knock-in were 14.0% and 20.6%, respectively; 67% of WT and 33% of the knock-in proteins had CV ≤ 15%. The latter can be explained by the fact that 425 proteins were differentially expressed in the knock-in mice (*P* < 0.00876, corresponding to *q* < 0.05). Within this group, 58 proteins were downregulated and 367 proteins were upregulated. Of the pituitary hormones detected, prolactin (PRL) was 17.1-fold lower in the *Lhx*3^*W*227*ter*/*W*227*ter*^ animals when compared to WT. Proopiomelanocortin (POMC, the precursor peptide containing ACTH) and *α*GSU also were significantly lower in the *Lhx*3^*W*227*ter*/*W*227*ter*^ mice, at 3.5-fold and 1.6-fold, respectively. LH*β* and TSH*β* were significantly higher in the *Lhx*3^*W*227*ter*/*W*227*ter*^ mice, at 1.8- and 1.7-fold. GH, the most abundant protein detected, was not significantly different between *Lhx*3^*W*227*ter*/*W*227*ter*^ and WT, whereas a negative control, ACTA1 (alpha actin), showed no significant change between the two groups. This investigation represents the most comprehensive proteomic analysis of the pituitary to date. This uniquely extensive dataset provides a resource for others investigating pituitary physiology and may serve to suggest biomarkers for future studies.

**Figure 5:**
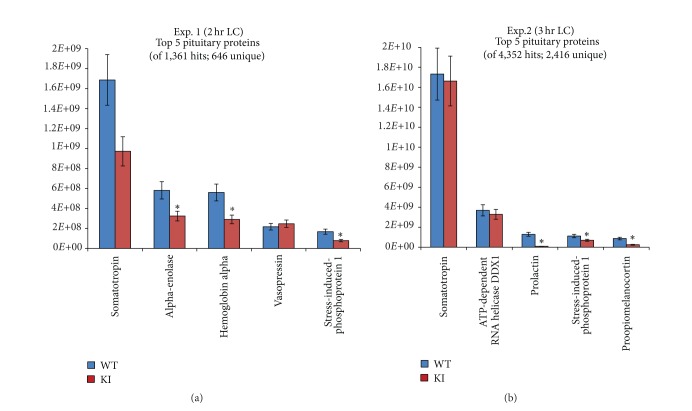
Impact of brain saline perfusion (Exp. 2) to reduce blood-protein contamination and expand the LC gradient by 50% (Exp. 2) on protein abundance among the top 5 pituitary proteins identified and quantified in the two experiments. WT: wild type mouse; KI: *Lhx*3^*W*227*ter*/*W*227*ter*^ knock-in mouse; *P < 0.00876, *q* < 0.05.

Not only did this investigation demonstrate the utility of the IdentiQuant LFQMS platform, but also the optimization of the approach demonstrated the critical importance of maximal LC conditions and proper sample preparation to accurate and reproducible protein quantitation. In an initial analysis of unperfused pituitary lysates from *Lhx*3^*W*227*ter*/*W*227*ter*^ and WT mice, a 2 h LC gradient was used. As Figure 5 illustrates, those conditions resulted in the identification of only 1,361 database hits and the identification and quantification of only 636 nonredundant proteins. An examination of the most abundant proteins detected revealed that hemoglobin alpha was the third-most abundant protein (along with albumin and several other serum proteins not shown), strongly suggesting substantial blood contamination. When new pituitary samples were dissected from brains perfused with ice cold saline (0.9%) and the LC gradient was expanded to 3 h, database hits increased 3.2-fold and nonredundant protein quantitation increased 3.7-fold. The blood-related proteins were not eliminated entirely, but their relative abundance was significantly reduced (<top 100 proteins). Perfusion and LC gradient expansion also increased the relative quantitation of the most abundant pituitary protein, somatotropin (growth hormone), along with several other hormones and pituitary proteins.

### 3.4. Cellular Membrane Proteomics

The IdentiQuantXL platform was recently used to verify the effectiveness and reproducibility of a novel membrane enrichment method [48]. Using HT29-MTX cells and a single cell disruption step (in a hypotonic reagent using liquid nitrogen), a single low speed centrifugation isolation step, and three wash steps using high speed centrifugation, the author identified and quantified 2,362 nonredundant proteins from an average yield of 237 *μ*g membrane proteins per 10 million cells. Across various experiments, 99% of the quantified proteins had a CV ≤ 30%, and 2,001 proteins (85%) had a CV ≤ 15%. Western blot and LC-MS/MS results of markers for cytoplasm, nucleus, mitochondria, and their membranes indicated that the enriched membrane fraction was highly pure by the absence of, or presence of trace amounts of, non-membrane marker proteins. These results not only document the effectiveness of the enrichment method, but also underscore the ability to comprehensively and accurately identify and quantify proteins from membrane fractions, a notoriously difficult task [49, 50].

## 4. Conclusion

Label-free mass spectrometric protein quantification based on peptide peak area is a viable alternative to array-based, gel-based, stable isotope tag or label-based, and spectral counting-based approaches. We have identified several issues associated with quantitation based on peptide ion peak area measurement: chromatographic alignment, peptide qualification for quantitation, and normalization. To overcome these issues, we incorporated several approaches into a label-free quantitative mass spectrometry platform, IdentiQuantXL. As the various applications of this platform demonstrate, comprehensive, accurate, and reproducible protein identification and quantitation are achievable in highly complex protein mixtures from experiments with many sample groups. 

## Figures and Tables

**Table 1:** Mean coefficient of variation across 2,282 Caco-2/HT29-MTX proteins in functionalized carbon nanotube exposures and LFQMS analysis.

Group	mean CV
Control	12.0%
SWCNT-COOH 500 pg/mL	25.0%
SWCNT-COOH 10 *μ*g/mL	14.9%
MWCNT-COOH 500 pg/mL	15.3%
MWCNT-COOH 10 *μ*g/mL	16.7%
MWCNT-PVP 500 pg/mL	16.5%
MWCNT-PVP 10 *μ*g/mL	21.3%

**Table 2:** Mean coefficient of variation across 4,112 Caco-2/HT29-MTX proteins in AgNP exposures and LFQMS analysis.

Group	3 h	24 h
Control	16.1%	17.1%
AgNP	19.1%	17.2%
